# Characterization of the pyruvate kinase gene family in soybean and identification of a putative salt responsive gene *GmPK21*

**DOI:** 10.1186/s12864-023-09929-7

**Published:** 2024-01-22

**Authors:** Wei Liu, Yubin Wang, Yanwei Zhang, Wei Li, Caijie Wang, Ran Xu, Haiying Dai, Lifeng Zhang

**Affiliations:** 1 Crop Research Institute, Shandong Academy of Agricultural Sciences, 202 Gongye North Road, Jinan, 250100 Shandong China; 2 Shandong Engineering Laboratory of Featured Crops, 202 Gongye North Road, Jinan, 250100 Shandong China

**Keywords:** Soybean, Pyruvate Kinase gene family, Genome identification, Salt stress Overexpression

## Abstract

**Background:**

As a key regulatory enzyme in the glycolysis pathway, pyruvate kinase (PK) plays crucial roles in multiple physiological processes during plant growth and is also involved in the abiotic stress response. However, little information is known about *PKs* in soybean.

**Results:**

In this study, we identified 27 *PK* family genes against the genome of soybean cultivar Zhonghuang13. They were classified into 2 subfamilies including PKc and PKp. 22 segmental duplicated gene pairs and 1 tandem duplicated gene pair were identified and all of them experienced a strong purifying selective pressure during evolution. Furthermore, the abiotic stresses (especially salt stress) and hormone responsive *cis*-elements were present in the promoters of *GmPK* genes, suggesting their potential roles in abiotic stress tolerance. By performing the qRT-PCR, 6 *GmPK* genes that continuously respond to both NaCl and ABA were identified. Subsequently, *GmPK21*, which represented the most significant change under NaCl treatment was chosen for further study. Its encoded protein GmPK21 was localized in the cytoplasm and plasma membrane. The transgenic *Arabidopsis* overexpressing *GmPK21* exhibited weakened salinity tolerance.

**Conclusions:**

This study provides genomic information of soybean *PK* genes and a molecular basis for mining salt tolerance function of *PKs* in the future.

**Supplementary Information:**

The online version contains supplementary material available at 10.1186/s12864-023-09929-7.

## Background

As an important metabolic pathway, glycolysis widely exists in higher plants and animals. Pyruvate Kinase (PK) is a key regulatory enzyme in the glycolysis pathway. It catalyzes the last step of glycolysis by transferring the high-energy phosphate group of phosphoenolpyruvates to adenosine diphosphate (ADP), and then produces ATP and pyruvate [1]. Pyruvate then acts as an important substance in organisms to participate in multiple metabolic reactions [2].

In plants, there are two isoenzymes of PKs in cytoplasm and plastid respectively, namely PKc and PKp [3]. They are different in physics, dynamics, immunology and expression regulation [3–7]. The PKs acting as PKc or PKp were found to be involved in multiple development and growth processes. It was reported that several *PK* genes in *Arabidopsis* were involved in oil and fatty acid biosynthesis in seeds [8, 9]. In tobacco, absence of leaf PKc resulted in a striking decrease in root biomass and root: shoot ratios [10]. Similarly, *GhPK6* in cotton played an important role in regulating cotton fiber elongation [11]. Compared with other plants, *PK* genes have been studied more systematically in rice. For instance, downregulation of *OsPK1* causes dwarfism and panicle enclosure in rice [12]; *OsPK5* is involved in rice glycolytic metabolism and GA/ABA balance for improving seed germination [13]. Furthermore, several *PK* genes were reported to be involved in the grain filling of rice [14–16].

With the development of transcriptomics and proteomics technology, the expression of *PK* genes or the accumulation of PK protein in plants suffering from abiotic stress were found to be affected. *AhABI4s* negatively regulate salt-stress response in peanut. The transcriptomics and quantitative proteomics analyses showed that PKs and other genes/proteins were affected by silencing of *AhABI4s* in peanut leaf and root after NaCl treatment. Furthermore, *AhABI4s* were able to bind to the promoters of pyruvate kinase (PK) coding genes in vitro [17]. In soybean, pyruvate kinase was increased under flooding, but gradually decreased during post-flooding recovery period at protein abundance, mRNA, and enzyme activity levels [18]. In pepper, the expression of *CaPKc1* gene was not only increased when the plants were infected by *Tobacco mosaic virus* (TMV), but also triggered by NaCl and wounding [19]. In *Brassica napus*, PKs showed an increased abundance under freezing stress after cold acclimation [20]. These reports suggest that *PKs* might be involved in the abiotic stress responses. However, the study of the *PKs* function in the abiotic stress tolerance is very limited. The only report is about a rice PK gene *OsPK5*. It could interact with OsSAP6, a member of stress-associated protein (OsSAP) gene family which could positively regulates saline-alkaline tolerance. Furthermore, overexpression of *OsPK5* in rice enhanced tolerance to soda saline-alkaline stress at seedling stage [21].

Soybean (*Glycine max* [L.] Merr.) is the leading oilseed crop produced and consumed in the world [22]. However, the production of soybean is severely threatened by the abiotic stresses, such as high salt [23]. Although the *PK* gene family has been identified and analyzed in *Arabidopsis* and rice [24, 25], little information is known about PK proteins in soybean. In this study, we performed genome-wide characterization of the soybean *PK* family genes against a reference soybean genome of a soybean cultivar, Zhonghuang13 [26] and analyzed their expression patterns under NaCl and ABA treatment. Furthermore, *GmPK21*, which was represented the most significant change under NaCl treatment was chosen for further study. The gene function analysis of *GmPK21* showed that it had a negative regulatory effect on salt stress tolerance in *Arabidopsis*. This research lays a foundation for further function investigations of *PK* genes in soybean.

## Results

### Identification and phylogenetic analysis of PK genes in soybean

We identified 27 soybean *PK* genes in this study (Table 1; Table S1). Those 27 soybean *PK* genes were named from *GmPK1* to *GmPK27*. The predicted protein products of these 27 *GmPK* genes varied from 184 (*GmPK3*) to 582 (*GmPK16* and *GmPK24*) amino acids. The protein MW (Molecular Weight) of these 27 GmPK proteins ranged from 20.06 kDa (GmPK3) to 63.68 kDa (GmPK20). Their predicted PI (isoelectric points) varied from 4.52 (GmPK3) to 8.01 (GmPK7). The protein sequences of 27 *GmPK* genes were shown in Table S1.

**Table 1:** List of pyruvate kinase family genes from soybean

Gene Name	Gene ID	Protein Length	Molecular weight (kD)	Isoelectric point
*GmPK1*	SoyZH13_01G183800.m1	545	60.28	7.69
*GmPK2*	SoyZH13_02G067000.m1	412	44.78	6.19
*GmPK3*	SoyZH13_02G067100.m1	184	20.06	4.52
*GmPK4*	SoyZH13_02G153600.m1	527	57.66	6.74
*GmPK5*	SoyZH13_03G170700.m1	527	57.66	7.09
*GmPK6*	SoyZH13_05G000800.m1	511	55.3	7.63
*GmPK7*	SoyZH13_05G076500.m1	265	29.49	8.01
*GmPK8*	SoyZH13_05G088000.m1	384	42.17	6.74
*GmPK9*	SoyZH13_07G210000.m1	501	54.29	7.66
*GmPK10*	SoyZH13_08G215000.m1	220	23.95	7.2
*GmPK11*	SoyZH13_09G112500.m1	577	63.34	7.65
*GmPK12*	SoyZH13_10G061700.m1	527	57.65	7.57
*GmPK13*	SoyZH13_10G163800.m1	569	62.88	6.01
*GmPK14*	SoyZH13_10G185200.m1	526	57.88	7.4
*GmPK15*	SoyZH13_10G209900.m1	543	59.79	7.01
*GmPK16*	SoyZH13_10G235900.m1	582	63.43	5.31
*GmPK17*	SoyZH13_11G041200.m1	310	34.92	6.56
*GmPK18*	SoyZH13_13G130400.m1	527	57.6	6.9
*GmPK19*	SoyZH13_16G135700.m1	502	54.76	6.36
*GmPK20*	SoyZH13_16G154700.m1	577	63.68	6.98
*GmPK21*	SoyZH13_19G000600.m1	510	55.24	7.63
*GmPK22*	SoyZH13_19G175100.m1	527	57.57	7.57
*GmPK23*	SoyZH13_20G023700.m1	502	54.34	7.22
*GmPK24*	SoyZH13_20G122100.m1	582	63.65	5.7
*GmPK25*	SoyZH13_20G150700.m1	575	63.17	7.29
*GmPK26*	SoyZH13_20G174800.m1	526	57.92	6.79
*GmPK27*	SoyZH13_20G196600.m1	567	62.52	6.21

**Fig. 1:**
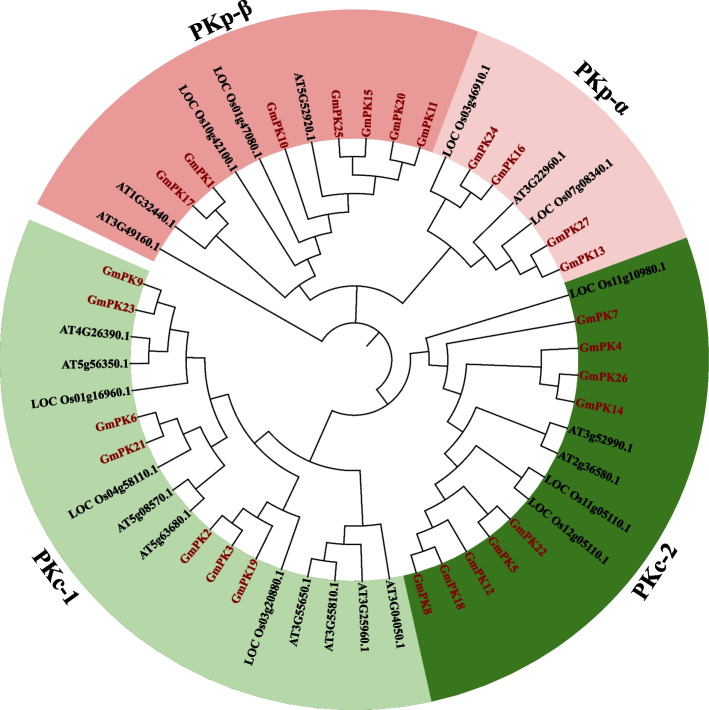
Phylogenetic tree based on protein sequences of *PK* genes in soybean, *Arabidopsis* and rice (At, *Arabidopsis thaliana*; Os, *Oryza sativa*)

We then built a phylogenetic tree by analyzing the amino acid sequences with the PK proteins from soybean, *Arabidopsis* and rice. Based on the phylogenetic analysis and previous research [24, 25], all of the PK proteins were divided into 2 subfamilies, PKc and PKp. The PKc subfamily was further divided into PKc-1 and PKc-2 clade. Similarly, the PKp subfamily was divided into PKp-α and PKp-β clade. As shown in Fig. 1, 11 GmPKs belonged to the PKp subfamily. Among them, 7 belonged to PKp-β clade and the other 4 belonged to PKp-α clade. The PKc subfamily contained 16 PKs. Of them, 7 were PKc-1 genes, and the other 9 were PKc-2 genes (Fig. 1).

### Chromosomal distributions, duplications and synteny analysis of the *GmPK* genes

**Fig. 2:**
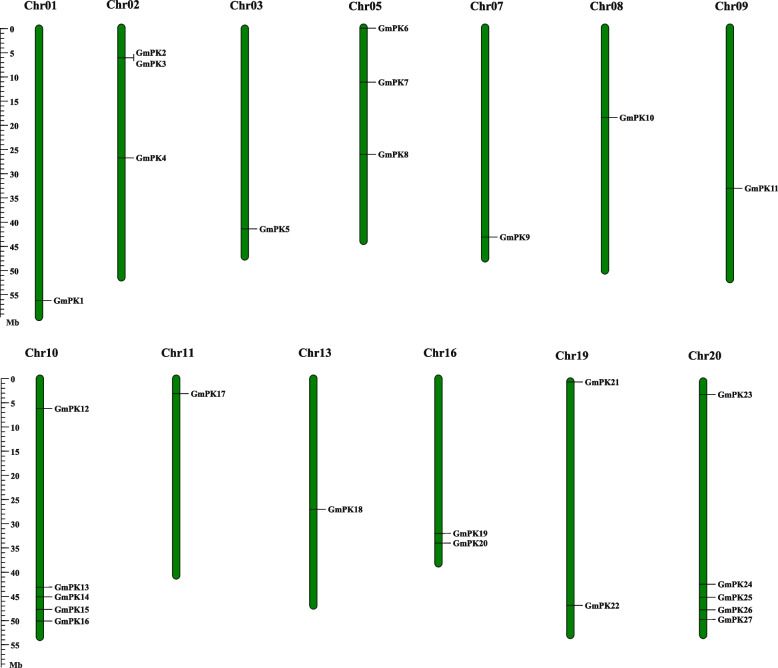
The chromosomal locations of *PK* genes in soybean. Green bars represent the 20 soybean chromosomes. The chromosome numbers are presented at the tops of the green bars. Scale bar on the left represents the lengths of each chromosome (Mb). Chr: Chromosome

These 27 *GmPK* genes were distributed on 13 out of the 20 chromosomes. The chromosome 10 (Chr 10) and 20 contained the largest number of *GmPK* genes (both of them had 5 *GmPK* genes), followed by Chr 2 and 5 each containing 3 *GmPK* genes. No *GmPK* genes were identified on Chr 4, 6, 12, 14, 15, 17 and 18 (Fig. 2).

**Fig. 3:**
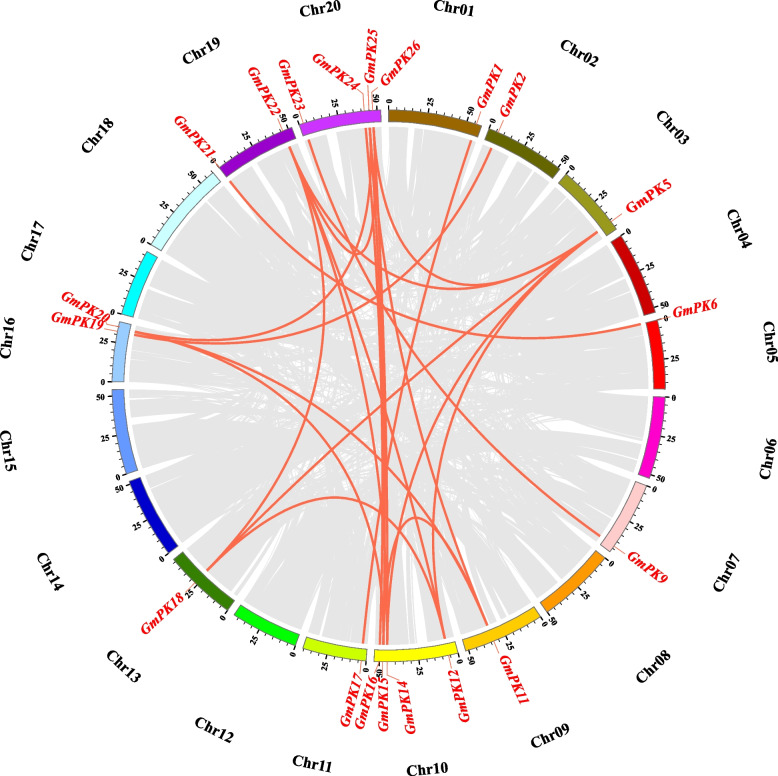
Circle plot of soybean chromosomes and the *GmPK* genes displayed as segmental duplicated gene pairs. The *GmPK* gene pairs are indicated by the red lines. Scale bar in marked on the chromosome indicates the chromosome lengths (Mb). Chr: Chromosome

Tandem and segmental duplications play important roles in the expansion of plant gene families [27]. To reveal the expansion mechanism of the *GmPK* gene family, the gene duplication events of the soybean *PK* genes were investigated. We found that only 1 pair of soybean *PK* genes was tandem repeat. 22 pairs of soybean *PK* genes were found to be segmental duplications (Fig. 3; Table S2). The nonsynonymous (Ka) and synonymous (Ks) substitution rates between these duplicated gene pairs were also calculated. The Ka/Ks of all the 23 duplicated gene pairs was found to be less than 1, suggesting that the soybean *PK* gene family might have experienced a strong purifying selective pressure during evolution (Table S2).

To detect the synteny of *GmPK* genes, a collinearity analysis between soybean and the other 2 plant species, including 1 monocot (rice) and 1 dicot (*Arabidopsis*) was performed. A total of 15 and 4 soybean *PK* genes showed a syntenic relationship with those in *Arabidopsis* and rice, respectively (Table S3). This finding suggests that soybean *PK* genes display a higher evolution divergence with monocotyledonous plants. Notably, we found 2 *GmPK* genes (*GmPK19* and *GmPK22*) were collinear with the *PK* genes from both of the *Arabidopsis* and rice (Table S3), suggesting that they might play important roles during the evolution of *GmPK* genes.

### Gene structure and conserved protein motif analysis of *GmPK* genes

**Fig. 4:**
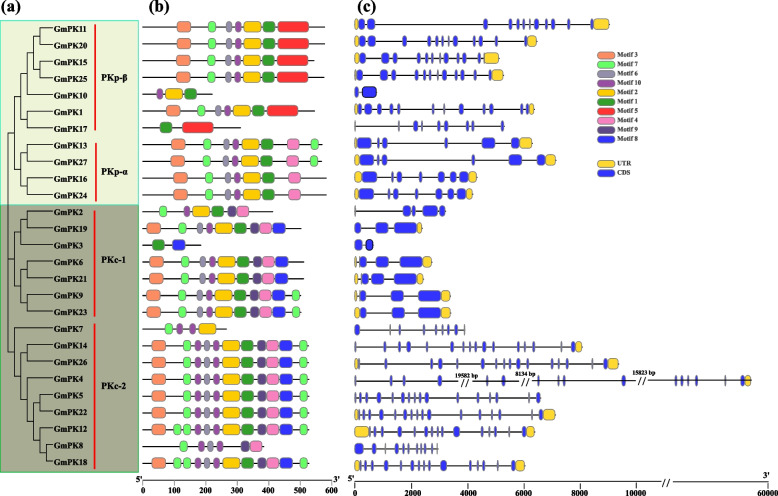
Conserved motifs and gene structures of *GmPKs*. **a** Phylogenetic tree based on protein sequences of *PK* genes in soybean. **b** Conserved motifs of GmPKs. The details of the motif were shown in Figure S2. **c** The gene structures of *GmPK* genes. CDS, introns, and untranslated regions (UTRs) are indicated by yellow boxes, black lines, and green boxes, respectively

By performing the multiple sequence alignment, we found that all of the 27 GmPKs shared the typical PK domain (Figure S1). Then the MEME program was used to detect conserved motifs in the *GmPK* gene family. 10 distinct motifs (named motif 1–10) were identified (Fig. 4b, Figure S2). Among them, Motif 1, 2, 3, 6, 7 and 10 belonged to the PK domain, motif 4 (or 5), 7 and 8 belonged to PK_C domain. In general, proteins from the same subfamily or clade were characterized by a similar motif type and distribution (Fig. 4a and b). Almost all of the PKc (including PKc-1 and PKc-2 clade) subfamily members (except GmPK2, GmPK3, GmPK7 and GmPK8) contained motif 3, 7, 6, 10, 2, 1, 9, 4 and 8. All the members in PKp-α clade contained the motif 3, 7, 6, 10, 2, 1 and 4. We also noticed that the motif 8 and 9 can only be detected in PKc subfamily, and motif 5 was only observed in the PKp-β clade.

In order to examine the gene structures, the exon–intron structures of the 27 *GmPK* genes were analyzed. As shown in Fig. 4c, the number of introns varied greatly between the clades of the same subfamily. The introns number of PKc-2 clade ranged from 8 to15. However, the introns number of PKc-1 clade members ranged from 1 to 4. Almost all of the *GmPK* genes in PKp-β clade contained 11 introns (except *GmPK10* and *GmPK17*), while the *GmPK* genes in PKp-α clade only contained 5 or 6 introns.

### Expression profiling of* PK* genes in different tissues of soybean

**Fig. 5:**
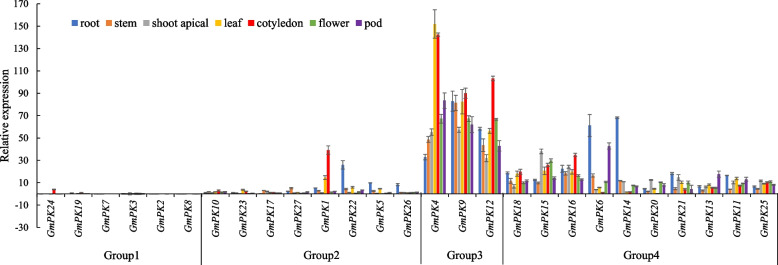
The expression profiles of the soybean *PK* genes in different tissues examined by qRT-PCR. The error bar represents the mean ± SD of three biological replicates

In order to investigate the transcript abundance of *PK* genes in different tissues, expression of the 27 *GmPK* genes covering 7 tissues of soybean was performed by qRT-PCR. Based on their expression pattern, the *GmPK* genes could be generally classified into 4 groups (Fig. 5). Genes in group 1 were barely detected in more than 5 soybean tissues. The genes in group 2 could be detected in more than 5 tissues, but the expression level was relative lower. There was a trend that all of those 3 genes in group 3 were highly expressed in all of the 7 tissues, and the 10 genes in group 4 were expressed with moderate level in almost all of the tissues (Fig. 5). Furthermore, several *GmPK* genes were found to display higher expression level in particular tissues. For example, *GmPK22*, *GmPK6*, *GmPK26* and *GmPK14* exhibited higher expression levels in roots than other tissues. The expression of *GmPK24*, *GmPK1* and *GmPK12* were relatively higher in cotyledon than in other tissues (Fig. 5).

### *Cis*-acting regulatory element analysis

**Fig. 6:**
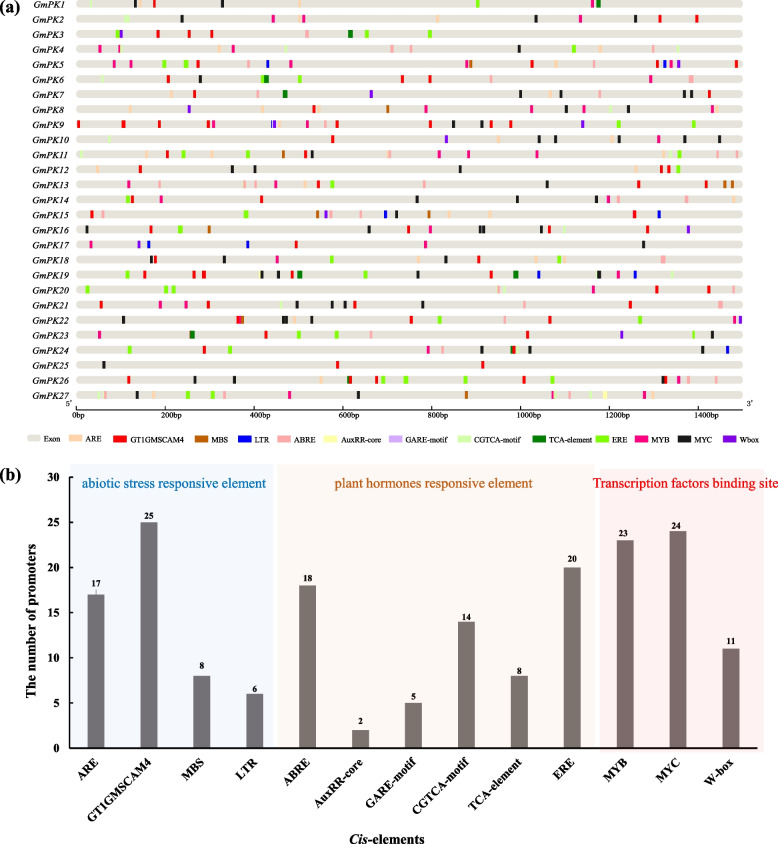
*Cis*-acting regulatory element analysis of *GmPK* genes. **a** Diagram of *cis*-acting regulatory elements in promoters of *GmPK* genes; **b** The distribution of the *cis*-elements related to abiotic stresses, plant hormones and transcription factors binding site. ARE: anaerobic responsive element; GT1GMSCAM4: salt-regulated element; MBS: drought responsive element; LTR: low temperature responsive element; ABRE: abscisic acid responsive element; AuxRR-core: Auxin response element; GARE-motif: gibberellin-responsive element; CGTCA-motif: methyl jasomonate (MeJA)-responsive element; TCA-element: salicylic acid responsive element; ERE: ethylene responsive element. W-box: WRKY binding site

To further understand the transcriptional regulation mechanisms of *GmPK* genes, we characterized the *cis*-acting regulatory elements within a 1500 bp upstream region from the transcription start site. As shown in Fig. 6 and supplementary Table S4, *cis*-elements related to abiotic stresses were identified, including the anaerobic responsive element (ARE), salt-regulated elements (SRE, GT1GMSCAM4), drought responsive elements (MYB binding site involved in drought-induction (MBS)) and low temperature responsive element (LTR). Several other regulatory elements associated with plant hormones were also detected, such as abscisic acid responsive element (ABRE), Auxin response element (AuxRR-core), gibberellin-responsive element (GARE-motif), methyl jasomonate (MeJA)-responsive element (CGTCA motifs), salicylic acid responsive element (TCA-element) and ethylene-responsive element (ERE). Of these abiotic stresses and hormone-responsive elements, the salt-regulated element (SRE, GT1GMSCAM4) was present in 25 out of the 27 promoters, being the most prevalent *cis*-element (Fig. 6b, Table S4). These results might imply that the *GmPK* genes could be regulated by hormones and abiotic stresses, especially the salt stress. Furthermore, the promoters of the *GmPK* genes contained predicted binding sites for several transcription factors, including MYB (23 out of 27 promoters), MYC (24 out of 27 promoters) and WRKY (11 out of 27 promoters) (Fig. 6; Table S4).

### Expression profiles of *GmPK* genes in response to salt stress and ABA treatment

**Fig. 7:**
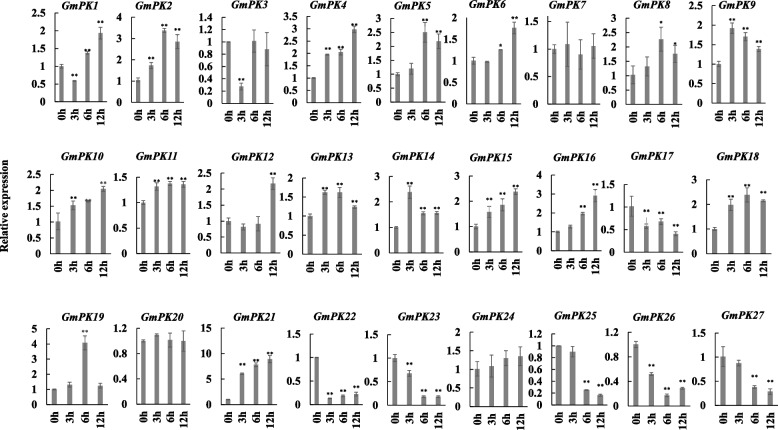
The expression of all the 27 *GmPK* genes under 200 mM NaCl treatment The error bar represents the mean ± SD of three biological replicates. Student’s t-test was used to examine the statistical significance (** *p* < 0.01, *p < 0.05)

In order to identify the salt-responsive *GmPK* genes in soybean, we performed qRT-PCR of all the 27 *GmPK* genes when the roots of soybean suffered from NaCl treatment at seedling stage. As shown in Fig. 7, the expression of 24 *GmPK* genes were found to be significantly changed in at least 1 time point. Among them, 16 genes were up-regulated by NaCl treatment, while 7 genes were down-regulated. 1 gene (*GmPK1*) was down-regulated at 3 h, but was up-regulated at 6 and 12 h. Notably, 15 *GmPK* genes were differently expressed at all of the 3 time-points (Fig. 7).

**Fig. 8:**
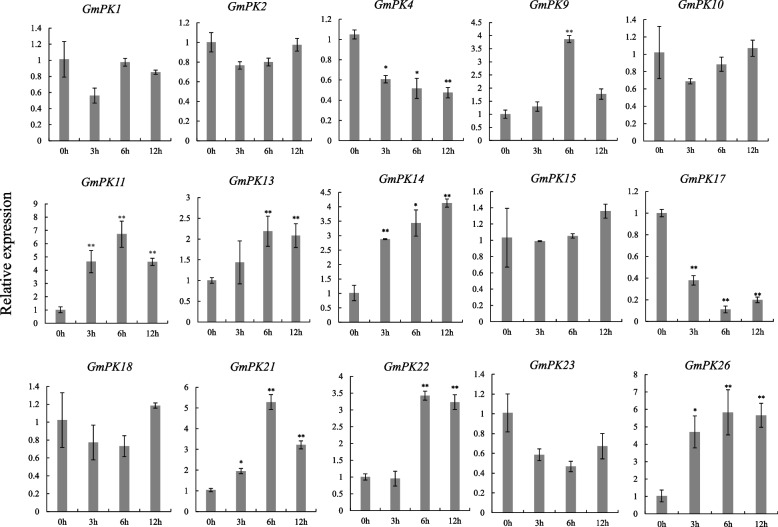
The expression of the 15 NaCl responsive *GmPK* genes under 100 µM ABA treatment. The error bar represents the mean ± SD of three biological replicates. Student’s t-test was used to examine the statistical significance (** *p* < 0.01, *p < 0.05)

We next examined the expression of these 15 *GmPK* genes which respond to NaCl at all of the 3 time point under 100 µM ABA treatment. The result displayed that the expressions of 6 *GmPK* genes (*GmPK4*, *GmPK11*, *GmPK14*, *GmPK17*, *GmPK21* and *GmPK26*) which were continuously regulated by salt were also significantly changed at all of the 3 time-points under ABA treatment (Fig. 8). Among them, the expression of *GmPK21* was represented the most significant change under NaCl treatment (Fig. 7), being the best candidate for further study.

### Expression of *GmPK21* and subcellular localization of its protein GmPK21

**Fig. 9:**
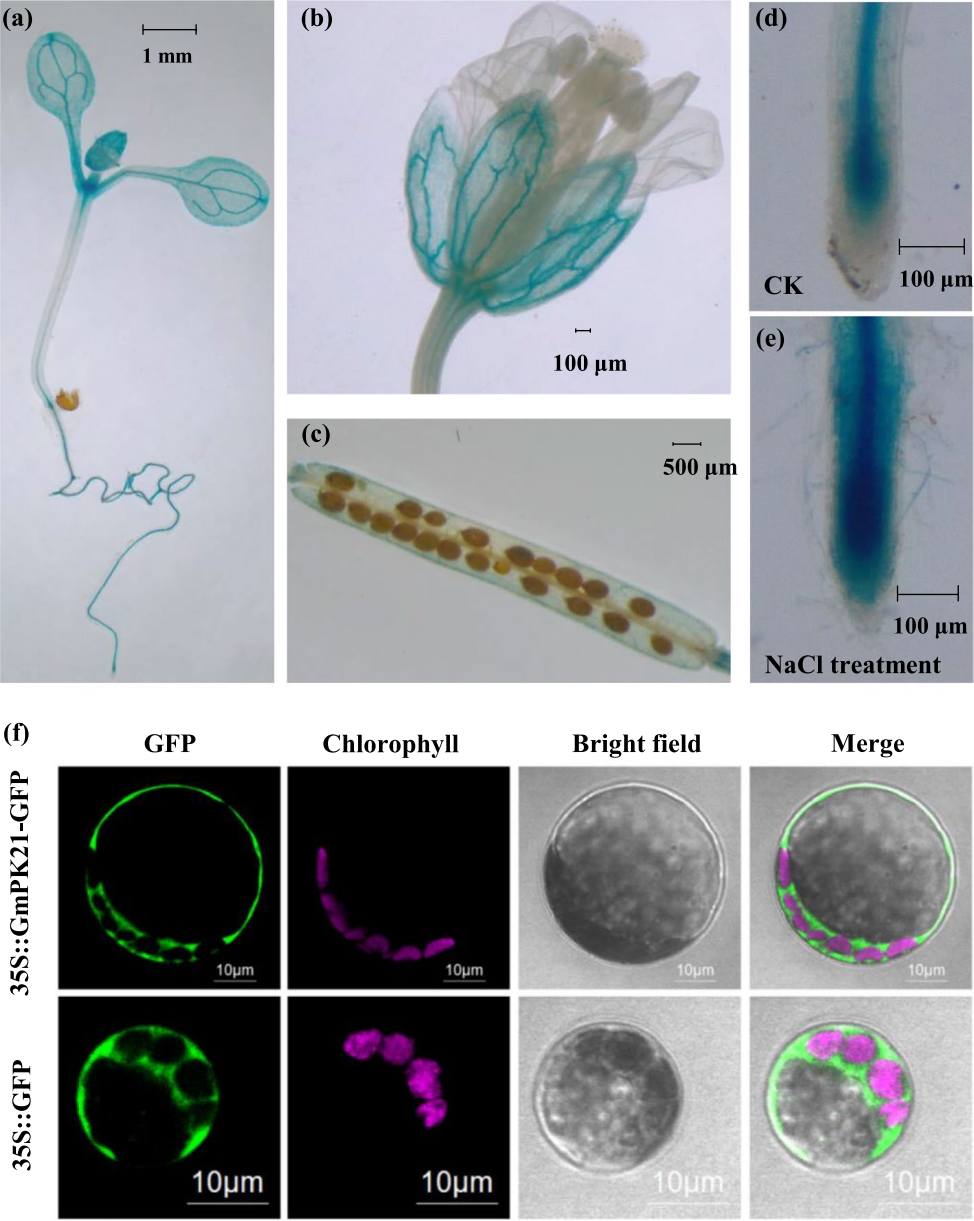
The expression pattern of *GmPK21* and subcellular localization of GmPK21. **a-c** The GUS activity in the different tissues of *GmPK21pro:GUS* transgenic *Arabidopsis*. **d** and **e** The GUS activity in the roots of *GmPK21pro:GUS* transgenic *Arabidopsis* treated by 100 mM NaCl for 6 h (**e**) and CK (**d**). **f** The subcellular localization of a GmPK21-GFP in *Arabidopsis* protoplasts

In order to clarify the details of the expression of *GmPK21*, the tissue expression pattern of *GmPK21* was analyzed by histochemical GUS staining of transgenic *Arabidopsis* in which the *GUS* gene was driven by the promoter of *GmPK21*. As shown in Fig. 9 a-c, the GUS signal could be detected in leaves, roots, shoot apical, calyx and silique, which is consistent with the expression pattern of *GmPK21* in different tissues of soybean measured by qRT-PCR (Fig. 5). Moreover, *GmPK21* was found to be highly expressed in vascular of the tested tissues. We then performed the GUS staining of the root when the plants were treated by 100 mM NaCl. The result showed that the GUS signal was significant stronger than control (Fig. 9 d-e), which is consist with the fact that *GmPK21* could be upregulated by NaCl treatment (Fig. 7).

In order to explore the subcellular localization of GmPK21, the GmPK21-GFP fusion protein was transformed into *Arabidopsis* protoplast (Fig. 9f) and tobacco protoplast (Figure S3). The result showed that the green fluorescent signal of GmPK21 was detected in the cytoplasm and plasma membrane (Fig. 9f, Figure S3).

### Ectopic expression of *GmPK21* in *Arabidopsis* reduced the plant salt tolerance

**Fig. 10:**
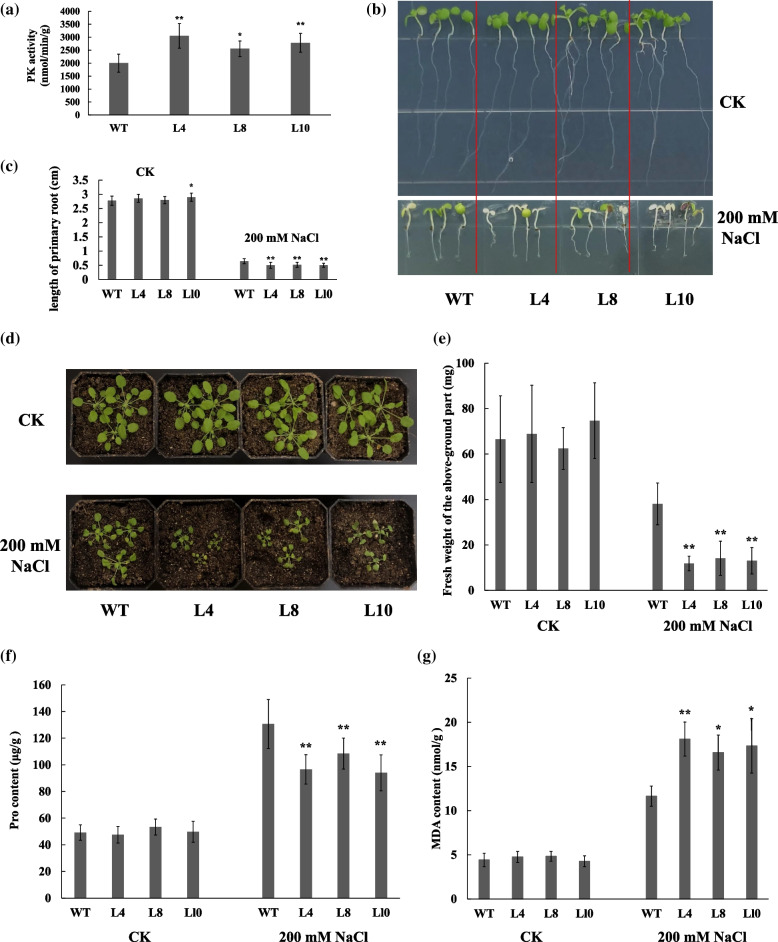
The phenotype of the 35S:*GmPK21* transgenic *Arabidopsis* plants under NaCl treatment. **a** The pyruvate kinase activity of GmPK21 in the wild type (WT) and the 35S:*GmPK21* transgenic *Arabidopsis* plants. **b** and **c** An overview (**b**) and the primary root length (**c**) of the wild type seedlings and the 35S:*GmPK21* transgenic *Arabidopsis* seedlings grown in 1/2 MS with or without 200 mM NaCl. **d** The phenotype of the WT and transgenic *Arabidopsis* grown in soil under normal condition or under 200 mM NaCl treatment. **e** The fresh weight of the WT and transgenic *Arabidopsis* grown under normal condition or under NaCl treatment. **f** The proline (Pro) and (**g**) malondialdehyde (MDA) contents of the transgenic and WT seedlings grown under normal condition or under 200 mM NaCl treatment. The data are means ± SD, and statistical significance was determined using Student’s t-tests: **, *P* < 0.01, *, *P* < 0.05. WT: the wild type *Arabidopsis*; L4, L8 and L10 represent 3 transgenic *Arabidopsis* lines overexpressed *GmPK21*

To investigate the role of *GmPK21* in salt tolerance, *GmPK21* was overexpressed in *Arabidopsis*. 3 transgenic lines displayed higher PK enzyme activity compared to WT were further used to perform NaCl treatment assay (Fig. 10a). The seedlings of the transgenic line 4 and 8 displayed similar growth status with wild type (WT), and the L10 showed slightly longer primary roots compared with WT on 1/2 MS medium (Fig. 10 b-c). However, when the 3-day-old seedlings were transfer to the 1/2 MS medium containing 200 mM NaCl, the growth status of all the 3 transgenic lines was weaker than WT, and the primary roots were shorter than WT (Fig. 10 b-c).

We further verified the salt tolerance of transgenic *Arabidopsis* in soil. The 21-day-old seedlings were subject to 200 mM NaCl for 19 days. The growth status and the fresh weights of the up-ground part of the seedlings were similar between the transgenic *Arabidopsis* and WT under normal condition. However, after 19 days NaCl treatment, the seedlings of transgenic lines grew significantly slower and weaker than WT, and the fresh weight of the aboveground parts of transgenic seedlings were much lower than that of WT (Fig. 10 d-e). The accumulations of proline could assist plants to resist the environmental stresses [28], and the MDA content is an important indicator of the degree of membrane lipid peroxidation and plasma membrane damage in plant cells. Therefore, we measured the Proline (Pro) and malondialdehyde (MDA) contents of the transgenic and WT seedlings. The Pro and MDA contents of all the seedlings didn’t show significant difference between transgenic *Arabidopsis* and WT under normal condition. However, 19 days after NaCl treatment, the accumulation of Pro and MDA in transgenic plants and WT was significantly promoted compared to that under normal condition. Moreover, the Pro content in transgenic *Arabidopsis* were significantly lower than that in WT seedlings, while the MDA content in transgenic *Arabidopsis* were significantly higher than that in WT (Fig. 10 f-g).

## Discussion

Pyruvate Kinase is a key regulatory enzyme in the glycolysis pathway. However, little information is known about *PK* genes in soybean. In this study, we identified 27 *PK* genes which were distributed in 13 out of the 20 chromosomes of soybean. The number of soybean *PK* genes is more than that in *Arabidopsis* (14), rice (10), potato (11) [29] and cotton (33) [11]. By examining the genome size and the density (number/Mb) of *PK* genes of the species above, we found that the density of *PK* genes in the soybean genome (0.03) is less than that in *Arabidopsis* (0.10) and potato (0.11), but similar to that in rice (0.03) and cotton (0.02). The differences in *PK* gene number and density between species might due to gene duplication events or the genome size [27, 30]. The soybean *PK* family genes were classified into 2 distinctive subfamilies PKc and PKp and each of them was further divided into two subclades. The result is consistent with the classification reported in *Arabidopsis* and rice [24, 25].

Gene duplication is one of the major evolutionary mechanisms for gene expansion [27, 31]. In general, gene families expand mainly by tandem and segmental duplications [27]. In our study, 22 pairs segmental duplicated gene (involved in 20 genes) representing 74.07% of the total 27 *GmPK* genes were identified. Therefore, the segmental duplication is the main mechanism of *GmPK* genes duplication. The soybean genome has undergone two rounds of whole genome duplication events, including the Legume WGD at around 59 million years ago (Ks < 0.3) and the Glycine WGD at around 13 million years ago (0.3 < Ks < 1.5) [32]. In the present study, half of the duplication events occurred during the Glycine genus WGD (whole-genome duplication) event, 8 gene pairs were separated during the legume WGD event. The duplication has contributed to adaptive evolution in plants [33]. Duplication gene pairs will experience different selection process, including subfunctionalization through purifying selective pressure (Ka/Ks < 1) [34] and neofunctionalization through positive selective pressure (Ka/Ks > 1) [35]. In soybean, most of the duplicated genes are subfunctionalized, only a small proportion of the duplicated genes have been neofunctionalized or nonfunctionalized [36]. In this study, all of the Ka/Ks ratios in different *PK* gene pairs were less than 1, suggesting that all of those duplicated *PK* genes were experienced a strong purifying selective pressure and subfunctionalized during evolution.

GmPK proteins from the same subfamily displayed similar motif type and distribution (Fig. 4). That is, the GmPKs of PKc-1 and PKc-2 had similar motif, and the GmPKs from PKp-α and PKp-β also had similar motif. However, the gene structures between each clade were different. The introns of PKc-2 genes are much more than those of PKc-1 genes, and the introns of PKp-β genes are much more than those of PKp-α genes (Fig. 4). This finding is consistent with that of the PKs in rice [25]. The results might indicate that the *PK* genes have changed the numbers and the length of introns to adapt to the environment during their evolutionary process but retained their conserved domains, which enabled the genes to perform their functions stably [25]. The genes expression patterns in different tissues provide clues to mining the potential function of these genes. In this study, a number of *GmPK* genes were constitutively expressed in most of the tested tissues, suggesting that they might play multiple roles during the development of soybean. In rice, a PKc-2 subclade gene *OsPK1* (*LOC_Os11g05110*) was reported to be involved in the plant morphological development. The *ospk1* mutant displayed later germination, shorter shoots, roots, leaves and internode and also showed marked reductions in seed number per panicle and seed set [12]. In the present study, 2 PKc-2 subclade member *GmPK4* and *GmPK12* were expressed in all of these tested tissues with a remarkable transcription level. This finding suggests that *GmPK4* and *GmPK12* might also be involved in the regulation of soybean morphological development. In *Arabidopsis, AtPKp1* (*At3g22960*) was reported to be involved in seed oil accumulation, embryo development and seed storage compounds mobilization upon germination [8, 9, 37]. Here, a soybean PKp gene *GmPK13* which is homologous to *AtPKp1* exhibited a higher expression in pod, suggesting its potential roles in the seed development.

Gene promoters are DNA sequences located upstream of gene coding regions and contain multiple *cis*-acting elements, which are specific binding sites for proteins involved in the initiation and regulation of transcription [38]. The promoters of *GmPK* were densed in transcription factors biding sites, including MYB, MYC and WRKY, indicating that these transcription factors may be relevant to *GmPK* expression. It was reported that the *cis*-acting element W-box (TTGACC) was present on the promoter of PK gene *DkPK1* in persimmon. By using the yeast one-hybrid method, DkWRKY3 and DkWRKY15 were found to interact with the promoter of *DkPK1* [39]. In this study, 11 *GmPK* genes possess the W-box (Fig. 6; Table S4) in their promoters. These *GmPK* genes probably could be upregulated by *WRKY* genes in soybean. Further study could use the yeast one-hybrid system to verify this speculation.

92.6% of the *GmPK* gene promoters contained the salt-regulated element (SRE, GT1GMSCAM4) (Fig. 6). Therefore, we speculated that *GmPK* genes might play roles on the soybean salt tolerance. However, studies on the involvement of soybean *PK* genes in salt stress response have rarely been reported. Based on the qRT-PCR, 16 *GmPK* genes were found to be up-regulated by NaCl (Fig. 7). Previously, Huang [40] screened a population of soybean accessions using GWAS analysis and identified QTL associated with salt tolerance. Within these QTL regions, 10 candidate genes including the *PK* gene *GmPK6* have been identified. In this study, the expression of *GmPK6* was induced at 6 and 12 h after NaCl treatment, indicating that *GmPK6* might be a candidate gene responsible for the salt tolerance. Furthermore, we identified 6 *GmPK* genes were continuously regulated by salt and ABA. It is noteworthy that 4 of them belonged to the PKc subfamily, suggesting that PKc subfamily might play important roles in the salt tolerance regulation. *GmPK21*, which showed the most sensitive to NaCl was selected to further analysis by ectopic expressed in *Arabidopsis*. The result showed that the *GmPK21*-overexpression plants were more sensitive to the salt stress, suggesting that *GmPK21* might negatively regulate salt tolerance. Notably, *GmPK21* is homologous to the rice *PK* gene *OsPK5* (LOC_Os04g58110) (Fig. 1). Zhu et al. reported that OsSAP6, a saline-alkaline tolerance regulator in rice could interacts with OsPK5. Furthermore, overexpression of *OsPK5* in rice improved soda saline-alkaline tolerance [21]. In our study, *GmPK21* is also involved in the salt stress tolerance regulation although it performed opposite function to its homologs in rice.This initial research on the function of *GmPK21* should be followed by further work that focuses on the soybean transformation, and then thoroughly explore its function on salt tolerance.

## Conclusions

This study provided a comprehensive characterization of the soybean *PK* family genes, highlighting their structures, expression patterns and the potential function in salt tolerance. The results indicated that *GmPK* genes could respond to the NaCl treatment. *GmPK21*, which represented the most significant change under NaCl treatment was found to negatively regulate the salinity tolerance. This research could have significant implications for the development of genetic improvement strategies to increase soybean tolerance to salt, thus contributing to food security and agricultural sustainability.

## Materials and methods

### Genome-wide identification of *PK* genes in soybean

All of the genome sequences data of soybean cultivar Zhonghuang13 were downloaded from the Genome Warehouse (GWH) database in the BIG Data Center under Accession Number GWHAAEV00000000.1 [26]. The Hidden Markow Model (HMM) of PK and PK_C domain (PF00224 and PF02887) was retrieved from the Pfam database (http://pfam.xfam.org) [41], and then was used to search against the Zhonghuang13 genome. All of the putative proteins were extracted and confirmed by the Pfam and NCBI-CDD database (https://www.ncbi.nlm.nih.gov/cdd). The information of soybean PK proteins including molecular weights and isoelectric points were calculated using the online program ExPASy (https://www.expasy.org) [42].

Phylogenetic tree was constructed using MEGA 7.0 by the neighbor-joining method (https://www.megasoftware.net) [43]. Bootstrap analysis was carried out with 1000 replications. The tree file was edited using ITOL v5 (https://itol.embl.de/).

### Chromosomal location and gene duplication analysis of soybean* PK* genes

The physical positions of soybean *PK* genes were extracted from the annotation file downloaded from GWH database. Then, the chromosomal location map was drawn using the MapChart (v.2.32) software [44]. Potential gene duplications were determined by two major criteria: length of aligned sequence covers ≥ 75% of longer gene and similarity of aligned regions is ≥ 75%. Ka and Ks values were calculated using KaKs Calculator (http://code.google.com/p/kaks-calculator/wiki/KaKs_Calculator) [45]. MCSscanX was used to detect the synteny of *PK* genes between soybean and other plants [46].

### Gene structure and conserved motifs analysis

The gene structures of *GmPK* genes were retrieved from the GFF3 annotation file downloaded from GWH database and then visualized by the Gene Structure Display Server (http://gsds.cbi.pku.edu.cn). To identify the conserved motifs of GmPK proteins, the online program MEME (http://meme-suite.org/tools/meme) was used.

### Expression analysis of soybean *PK* genes in different tissues

Soybean cultivar Zhonghuang13 was grown in a controlled culture room under short day condition (12 h light/12 h dark). The root, stem, leaf, shoot apical, cotyledon was sampled at 12 days after emergence (DAE), and the flower and pod were sampled at 34 DAE and 50 DAE, respectively. Each sample contained 3 whole roots from 3 independent plants. Three biological replicates were performed. The total RNA of these samples was extracted and reverse-transcribed for subsequent quantitative real-time PCR (qRT-PCR) analysis. Data were presented as means ± SD, and Student’s t-tests were used by SPSS statistics 19 to assess the significance of differences.

### *Cis*-element analysis of *GmPK* genes

For the *cis*-element analysis, the 1500 bp upstream sequences of soybean *PK* family genes were obtained from the GWH database. The PLANTCARE database (http://bioinformatics.psb.ugent.be/webtools/plantcare/html/) was used to predict the *cis*-elements of the *GmPK* gene promoters.

### Identification of soybean *PK* genes in response to the NaCl and ABA treatment

The seedlings of soybean cultivar Zhonghuang13 were grown in a controlled culture room at 25 °C with the photoperiod of 12 h light/12 h dark. For the NaCl and ABA treatment, 14-day-old seedlings were gently removed from the soil and cultivated in Hoagland liquid medium for 2 days. After that, the seedlings were transferred into new Hoagland liquid medium containing 200 mM NaCl or 100 μM ABA. The roots from control and treated seedlings were sampled at 0 h, 3 h, 6 h and 12 h after treatment and then immediately immersed in liquid nitrogen and stored at -80 °C for RNA extraction and qRT-PCR. Each sample contained 3 whole roots from 3 independent plants. Three biological replicates were performed. Data were presented as means ± SD, and Student’s t-tests were used by SPSS statistics 19 to assess the significance of differences.

### qRT-PCR analysis

The total RNA of the tested samples was extracted using Trizol reagent. qRT-PCR with 3 technical replicates for each of the triplicate biological samples was conducted to measure the expression levels of the *GmPK* genes. qRT-PCR was performed by using Roche 480 Light Cycler (Roche, Mannheim, Germany) and the Takara SYBR Premix Extaq (Takara, Japan). Gene expression levels were then calculated according to the method (2^−ΔΔCT^) described by Livak and Schmittgen [47]. For the expression pattern of those 27 *GmPK* genes in different tissues and under NaCl treatment, *GmActin* (Glyma.18G290800) was used as the reference gene. For the expression level of *GmPK21* in the transgenic *Arabidopsis*, *AtActin2* (AT3G18780) was used as the reference gene. The details of the primers were shown in Table S5.

### Histochemical GUS assays

The promoter of *GmPK21* was amplified by genomic PCR (The primers *GmPK21*pro-F and *GmPK21*pro-R were shown in Table S5) and then was inserted into the linearized DX2181 vector (digested by HindIII and BamHI) using the EasyGeno Assembly Cloning kit (Tiangen, VI201), generating the *GmPK21*pro-GUS fusion plasmid. The plasmid was then introduced into the *Agrobacterium tumefaciens* strain GV3101 and then transferred to *Arabidopsis* ecotype Columbia by using the floral dip method [48]. To detect GUS activity in different tissues of transgenic *Arabidopsis*, the seedlings, flowers and siliques were sampled and incubated in the X-Gluc solution for 16 h at 37 °C [49]. To detect the GUS activity after the NaCl treatment, the *Arabidopsis* seedlings were submerged in the 1/2 MS medium containing 100 mM NaCl. The roots of the *Arabidopsis* treated by NaCl were sampled after 6 h treatment, and the roots of the *Arabidopsis* which submerged in the 1/2 MS medium without NaCl were used as control. Then all of the samples were incubated in the X-Gluc solution for 16 h at 37 °C. In order to remove the chlorophyll, the samples were transferred into the ethanol (70% v/v) and then observed under stereomicroscope (Leica S9i, Germany). The samples were collected from 3 different transgenic *Arabidopsis* lines.

### Subcellular localization of GmPK21

The CDS without the termination codon of *GmPK21* was amplified by PCR using the primer *GmPK21*-F1 and *GmPK21*-R1 (Table S5) and then was introduced into the plasmid linearized pAN580 vector (digested by XbaI and BamHI) using the EasyGeno Assembly Cloning kit (Tiangen, VI201), generating the *GmPK21*-GFP fusion plasmid. The empty vector pAN580 was used as the control. The *GmPK21*-GFP and empty vector pAN580 were transformed into the *Arabidopsis* or tobacco protoplasts [50] respectively. The GFP signal was then analyzed using the LSM710 confocal microscope (Zeiss, Oberkochen, Germany).

### Overexpression of *GmPK21* in *Arabidopsis*

The full length of the *GmPK21* CDS was amplified by PCR using the primer *GmPK21*-F2 and *GmPK21*-R2 (Table S5) and then was introduced into the linearized plasmid pC3300s (digested by SacI and BamHI) using the EasyGeno Assembly Cloning kit (Tiangen, VI201), generating the pC3300s-35S::*GmPK21*. The construct was then transformed into *Arabidopsis* (Col-0) using Agrobacterium GV3101. The seeds of transformants were selected by 1/2 MS medium containing glufosinate solution. The RT-PCR and qRT-PCR was then performed to confirm the presence of the *GmPK* gene in the glufosinate resistant lines (The RT-PCR primers RT-*GmPK21*-F/ RT-*GmPK21*-R and qRT-PCR primers q*GmPK21*-F (Ara)/q*GmPK21*-R(Ara) were shown in Table S5). The PK activity of 20-day-old transgenic *Arabidopsis* and WT was determined by using the Pyruvate Kinase (PK) Activity Assay Kit (BC0540, Solarbio life sciences). 3 biological replicates were performed and 10 seedlings (aboveground parts) were pooled per biological replicate. Data were presented as means ± SD, and Student’s t-tests were used by SPSS statistics 19 to assess the significance of differences.

### Salt stress treatment of the *GmPK21* transgenic *Arabidopsis*

Three lines of the T3 transgenic *Arabidopsis* plants were used for NaCl treatment assay. The *Arabidopsis* were germinated in the 1/2 MS medium. The 3-day-old seedlings were then transferred to the new 1/2 MS medium containing 200 mM NaCl. The seedlings in the 1/2 MS without NaCl were used as control. 6 days later, the status of the plants was observed and the length of the primary roots were measured. At least 20 seedlings’ roots were measured for each line. Data were presented as means ± SD, and Student’s t-tests were used by SPSS statistics 19 to assess the significance of differences.

Meanwhile, 3-day-old seedlings were transferred to the soil, and grown in an incubator (23℃) under short day condition (8 h light/16 h dark). 21 days later, seedlings were treated with 200 mM NaCl for 19 days. The status of the plants was observed and the fresh weight of aboveground parts of the seedlings were measured. At least 20 seedlings’ fresh weight were measured for each line. Data were presented as means ± SD, and Student’s t-tests were used by SPSS statistics 19 to assess the significance of differences. Then the aboveground parts of the *Arabidopsis* seedlings were used to measure the proline (Pro) and malondialdehyde (MDA) content. The content of Pro and MDA was determined by Proline (Pro) Content Assay Kit (Solarbio, BC0290) and Malondialdehyde (MDA) Content Assay Kit (Solarbio, BC0025) cording to the kit instructions. 3 biological replicates were performed and 10 seedlings were pooled per biological replicate.

### Supplementary Information

**Additional file 1: Figure S1.** Multiple sequence alignment of 27 GmPKs. The red and blue bars represent the PK domain and PK_C domain, respectively.

**Additional file 2: Figure S2.** The consensus sequences and their logos of the 10 conserved motifs predicted by MEME program.

**Additional file 3: Figure S3.** The subcellular localization of a GmPK21-GFP in tobacco protoplasts.

**Additional file 4: Table S1.** Sequences of PK genes in soybean, *Arabidopsis* and rice.

**Additional file 5: Table S2.** Segmental and tandem duplications of *GmPK* gene pairs in soybean and inference of duplication time.

**Additional file 6: Table S3. **Synteny analysis of *PK *genes between soybean and other organism (*Arabidopsis* and Rice).

**Additional file 7: Table S4.** *Cis*-regulatory element analysis in soybean *PK* genes.

**Additional file 8: Table S5.** Sequences of primers used in this study.

## Data Availability

All of the sequences data of soybean cultivar Zhonghuang13 were downloaded from the Genome Warehouse (GWH) database in the BIG Data Center under Accession Number GWHAAEV00000000.1 (https://ngdc.cncb.ac.cn/soyomics/download). The CDS and protein sequences of *GmPKs* were shown in Table S1. The protein sequences of PK genes in *Arabidopsis* and rice were obtained from the database Phytozome (https://phytozome-next.jgi.doe.gov/) and were shown in Table S1.
