# Supplementary material for: Characterization of the pyruvate kinase gene family in soybean and identification of a putative salt responsive gene GmPK21
Source: BMC Genomics. 2024 Jan 22;25:88. doi: 10.1186/s12864-023-09929-7 (PMC10802038; doi:10.1186/s12864-023-09929-7)

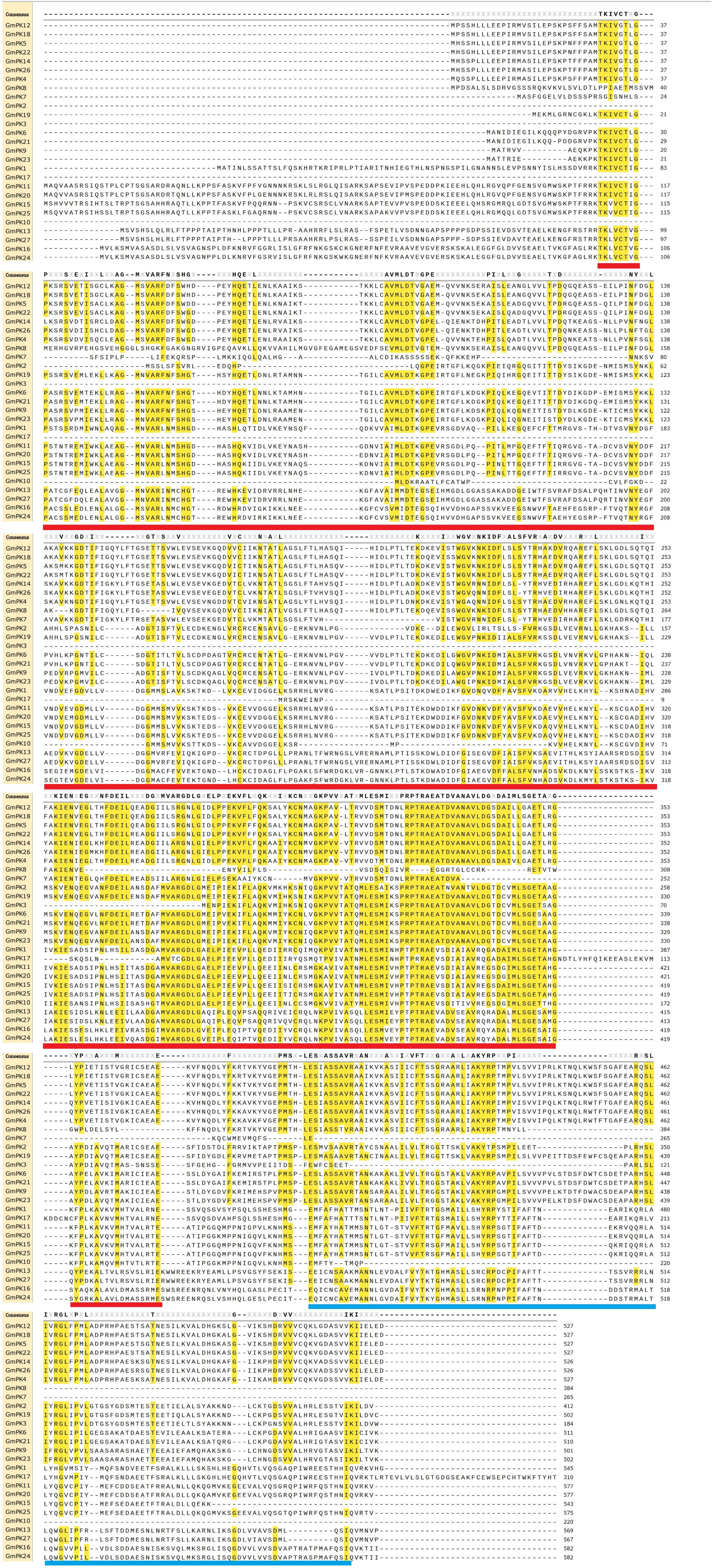

Supplement: Supplementary file 1 — Additional file 1: Figure S1. Multiple sequence alignment of 27 GmPKs. The red and blue bars represent the PK domain and PK_C domain, respectively. [file 12864_2023_9929_MOESM1_ESM.pdf]
