# Supplementary figures and images for: Characterization of the pyruvate kinase gene family in soybean and identification of a putative salt responsive gene GmPK21
Source: BMC Genomics. 2024 Jan 22;25:88. doi: 10.1186/s12864-023-09929-7 (PMC10802038; doi:10.1186/s12864-023-09929-7)

*35S::GmPK21-GFP*

GFP

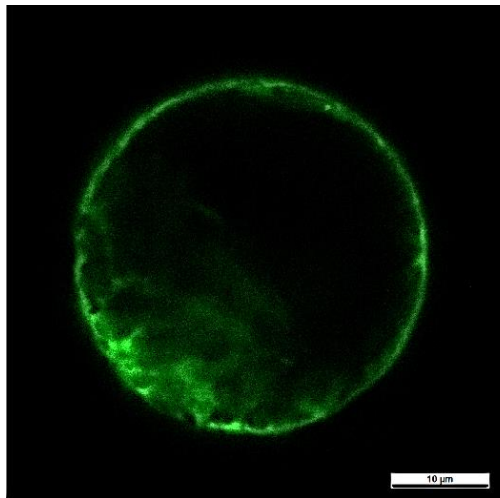

Chlorophyll fluorescence

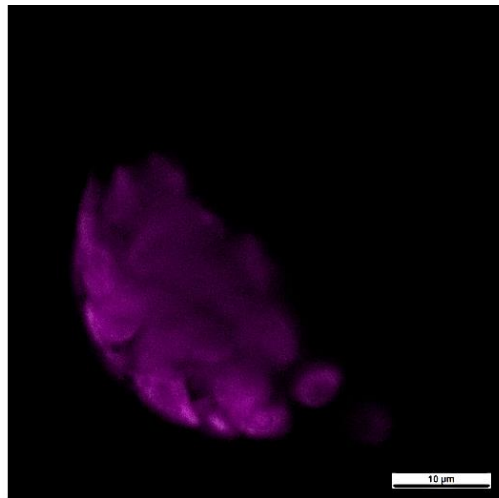

Bright Field

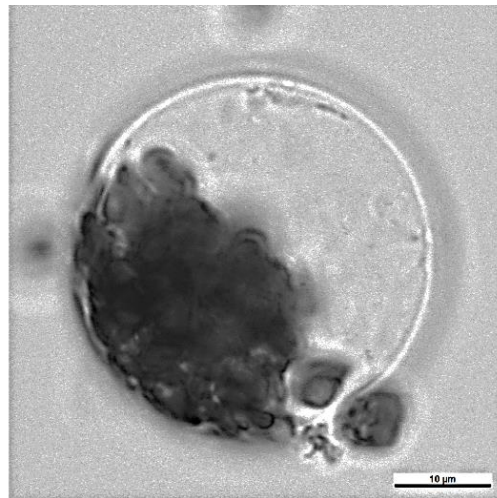

Merged

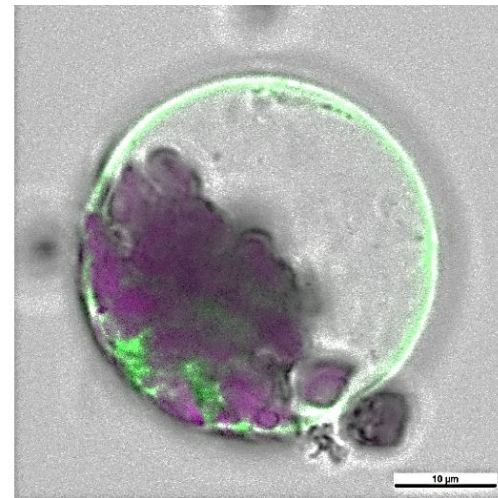

*35S::GFP*

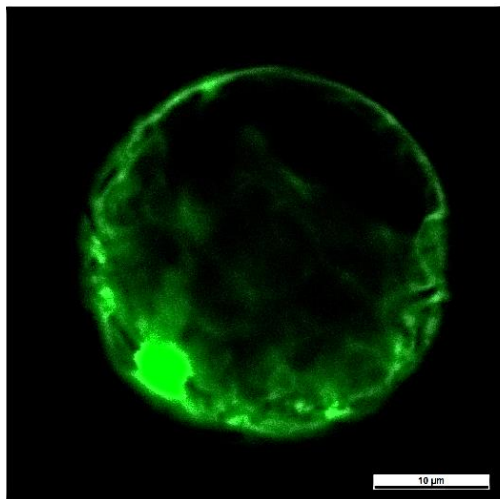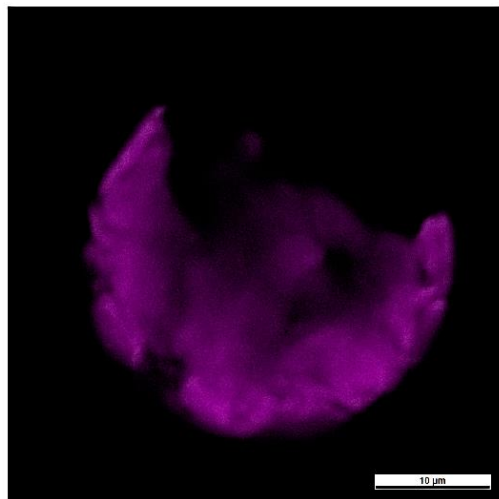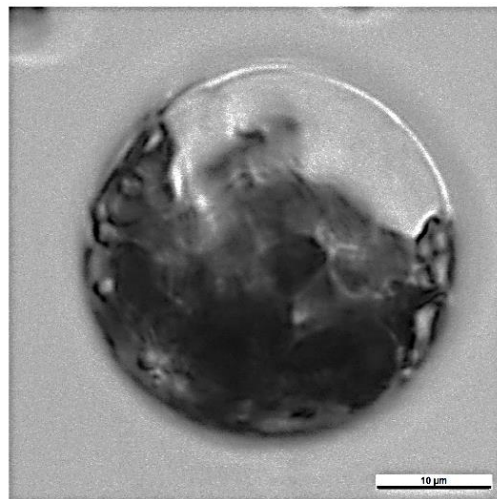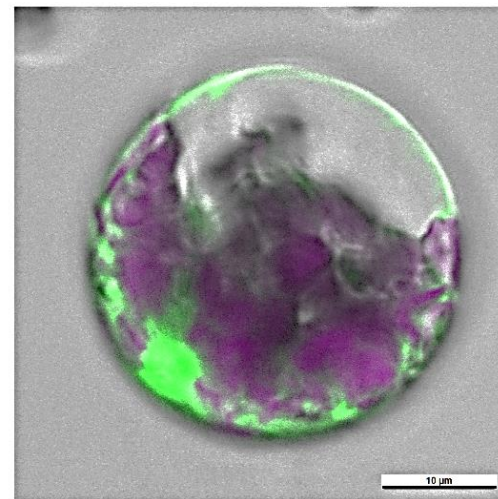

Supplement: Supplementary file 3 — Additional file 3: Figure S3. The subcellular localization of a GmPK21-GFP in tobacco protoplasts. [file 12864_2023_9929_MOESM3_ESM.pdf]
